# Substrates (Acyl‐CoA and Diacylglycerol) Entry and Products (CoA and Triacylglycerol) Egress Pathways in DGAT1

**DOI:** 10.1002/jcc.70108

**Published:** 2025-04-19

**Authors:** Hwayoung Lee, Wonpil Im

**Affiliations:** ^1^ Department of Biological Sciences Lehigh University Bethlehem Pennsylvania USA

**Keywords:** acyl‐CoA, DGAT1, diacylglycerol, diacylglycerol O‐acyltransferase 1, MBOAT, triacylglycerol

## Abstract

Diacylglycerol O‐acyltransferase 1 (DGAT1) is an integral membrane protein that uses acyl‐coenzyme A (acyl‐CoA) and diacylglycerol (DAG) to catalyze the formation of triacylglycerides (TAGs). The acyl transfer reaction occurs between the activated carboxylate group of the fatty acid and the free hydroxyl group on the glycerol backbone of DAG. However, how the two substrates enter DGAT1's catalytic reaction chamber and interact with DGAT1 remains elusive. This study aims to explore the structural basis of DGAT1's substrate recognition by investigating each substrate's pathway to the reaction chamber. Using a human DGAT1 cryo‐EM structure in complex with an oleoyl‐CoA substrate, we designed two different all‐atom molecular dynamics (MD) simulation systems: DGAT1^away^ (both acyl‐CoA and DAG away from the reaction chamber) and DGAT1^bound^ (acyl‐CoA bound in and DAG away from the reaction chamber). Our DGAT1^away^ simulations reveal that acyl‐CoA approaches the reaction chamber via interactions with positively charged residues in transmembrane helix 7. DGAT1^bound^ simulations show DAGs entering into the reaction chamber from the cytosol leaflet. The bound acyl‐CoA's fatty acid lines up with the headgroup of DAG, which appears to be competent to TAG formation. We then converted them into TAG and coenzyme (CoA) and used adaptive biasing force (ABF) simulations to explore the egress pathways of the products. We identify their escape routes, which are aligned with their respective entry pathways. Visualization of the substrate and product pathways and their interactions with DGAT1 is expected to guide future experimental design to better understand DGAT1 structure and function.

## Graphical abstract

Two distinct substrate pathways of diacylglycerol O‐transferase 1 (DGAT1) were investigated using molecular dynamics simulation. The study revealed that each substrate preferentially utilizes a different segment of the protein complex as its primary entry route into the reaction chamber. In the presence of Acyl‐CoA, diacylglycerol primarily approached the TM1‐IL2 gap, whereas Acyl‐CoA accessed the reaction chamber through the TM7‐TM8 gap in the absence of diacylglycerol. The products of the enzymatic reaction (triacylglycerol and CoA) utilize the same pathway for egress from the complex.

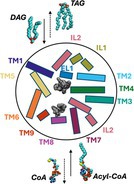

## 1 Introduction

A proper storage of energy reserves is vital for the survival of all living organisms, serving as a preparation for potential future challenging conditions. Triacylglycerols (TAGs) represent the most efficient and commonly utilized form of energy storage, consisting of three acyl chains esterified to a glycerol backbone [1, 2]. TAGs are generally manufactured in the endoplasmic reticulum (ER) of the liver and adipose tissues via a series of steps referred to as the Kennedy pathway [3, 4]. In the final phase of this pathway, diacylglycerol O‐acyltransferase 1 (DGAT1) is responsible for an enzymatic reaction to convert diacylglycerol (DAG) and acyl‐CoA into TAG and CoA by transferring an acyl group from acyl‐CoA to the *sn*‐3 position of DAG. Such a TAG production can then be stored in lipid droplets for specific functions [4, 5, 6].

Elevated levels of TAGs are associated with metabolic disorders such as cardiovascular disease and diabetes, often connected to obesity and dysregulated TAG metabolism in humans [7, 8]. Given its essential function in lipid metabolism and its importance for numerous physiological activities, interest in DGAT1 has significantly increased in recent years. In addition to a crucial energy storage system, TAG production enables cells to handle surplus free fatty acids and avoid their accumulation to dangerous levels [9, 10, 11, 12, 13]. Therefore, by promoting TAG production, DGAT1 ensures that cells can securely store these fatty acids and utilize them when necessary, preserving lipid equilibrium and preventing metabolic disorders. Also, DGAT1 is required for liver metabolism, particularly concerning nonalcoholic fatty liver disease, which is marked by an excessive buildup of fat in liver cells.

Beyond its function in lipid metabolism, DGAT1 is involved in various physiological and pathological processes as well. In terms of cardiovascular diseases, an excessive accumulation of lipids, especially TAGs, can lead to issues such as atherosclerosis and other circulatory ailments. An imbalance in DGAT1 activity may worsen the accumulation of lipids in blood vessels, heightening the risk of plaque development and resulting in inflammation and damage to the vascular system [8]. Additionally, various cancers exhibit elevated production of TAGs, indicating a potential influence on the survival and proliferation of cancer cells [14, 15]. These various roles of DGAT1 indicate why it has gained significant interest from researchers recently. It acts as an essential link between energy storage, lipid metabolism, and the regulation of numerous cellular processes.

DGAT1 belongs to the MBOAT (membrane‐bound O‐acyl transferase) family, a diverse collection of enzymes present in all living organisms [16, 17, 18]. In the human genome, there are 11 unique MBOAT enzymes, each with its own specialization in acylating various lipids or proteins [19, 20, 21]. These enzymes, including DGAT1, share a common structural framework with 9–12 transmembrane (TM) domains within organelle membranes such as the ER. MBOAT enzymes have specific cavities or pockets known as reaction chambers, which are crucial for substrate binding and enzymatic processes. These cavities enable the enzymes to accommodate substrates of varying molecular shapes and sizes, ensuring specificity in the acylation process.

**FIGURE 1:**
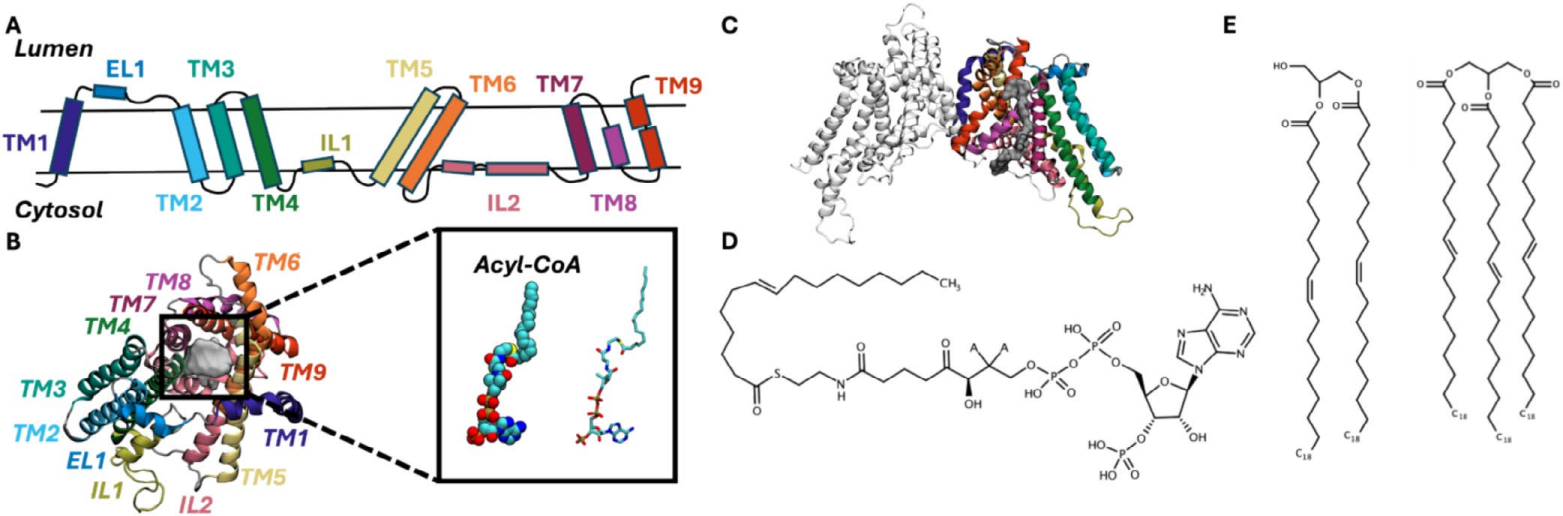
Cartoon and chemical structure representations of DGAT1, DAG, acyl‐CoA, and TAG. (A) Topology of DGAT1. Each secondary structure is colored differently. Note that the cytosolic side is always in the lower leaflet side in all membrane‐containing figures in this work. (B) Top view of the reaction chamber from the ER lumen side with acyl‐CoA (gray density) bound. (C) Side view of DGAT1 dimeric complex. (D, E) Chemical structures of (D) acyl‐CoA and (E) DAG and TAG used in this study.

The single‐particle cryo‐electron microscopy (cryo‐EM) technique has been used to resolve the structure of dimeric human DGAT1 (Figure 1) [22, 23]. An oleoyl‐CoA is observed in the reaction chamber within the membrane hydrophobic core. The oleoyl‐CoA, positioned near the HIS415 residue (a critical residue for the enzymatic reaction) [16, 17, 18, 19, 20, 21, 22, 23], exhibits a curved acyl tail conformation and occupies a substantial opening between TM7 (399–424), TM8 (428 to 447), and TM9 (450–481). Furthermore, there is an additional lateral opening on the opposite side of the protein, adjacent to TM1 (85–111), EL1 (112–130), and TM5 (274–307), mostly embedded in the bilayer membrane as part of the protein's outermost side on the cytosolic side, which might be used for DAG entry and TAG egress (Figure S1). Although DGAT1‐substrate binding has been explored using experimental techniques, to the best of our knowledge, there are no simulation studies to investigate the interactions between DGAT1 and its substrates and products, as well as their entry and egress pathways. In this study, we have used all‐atom molecular dynamics (MD) simulations to explore intricate interactions between the DGAT1 dimeric complex and its substrates (DAG and acyl‐CoA) and products (TAG and CoA), as well as their entry and egress pathways.

## 2 Methods

### 2.1 Simulation System Details

**FIGURE 2:**
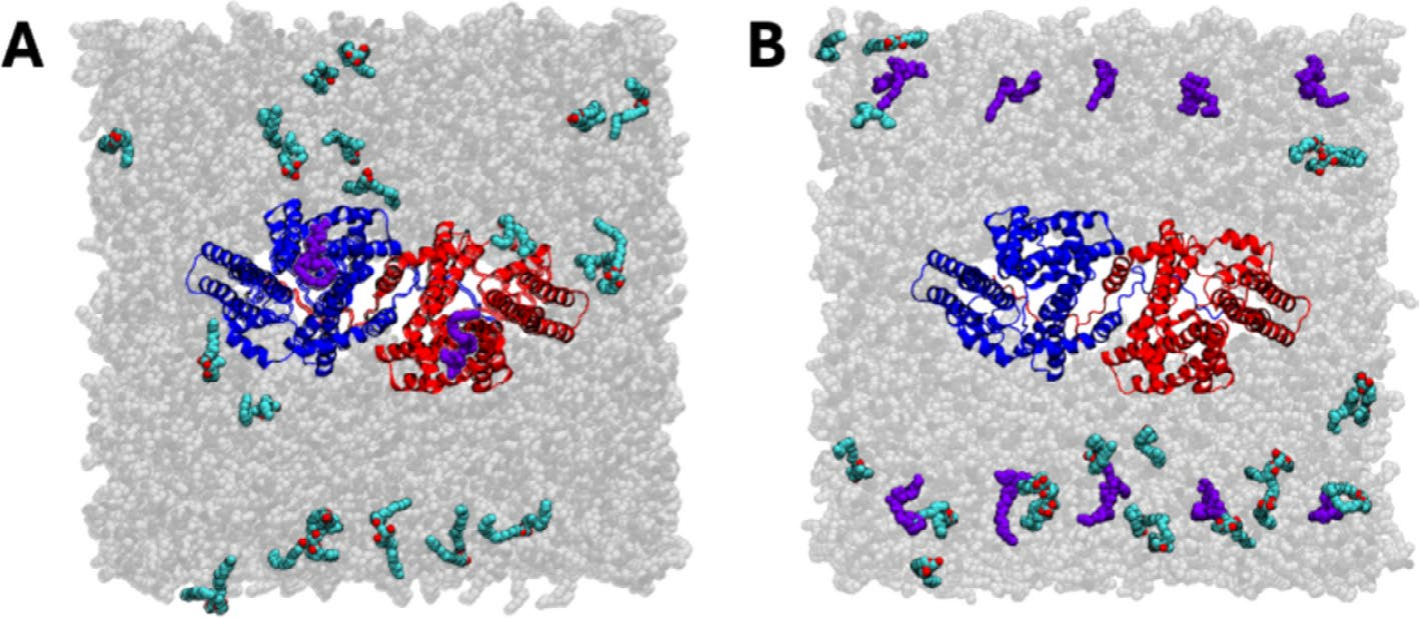
Snapshots of initial simulation systems. (A) DGAT1^bound^ with acyl‐CoA located at the reaction chamber of each protomer, and 10 DAGs freely distributed on the bilayer membrane. (B) DGAT1^away^ with both substrates (10 acyl‐CoA and 10 DAG molecules) located away from DGAT1. Protomers A and B are colored in blue and red, respectively. DAG is colored cyan (tails) and red (headgroup), and acyl‐CoA is colored purple. Ions and water are omitted for clarity.

To investigate the entry pathways of two substrates (DAG and acyl‐CoA) to DGAT1, two sets of all‐atom MD simulation systems (Table 1) were prepared with the cryo‐EM DGAT1 dimer structure (PDB ID: 6VP0) [22]. First, 1,2‐dioleoyl‐3‐phosphatidylcholine (DOPC), 1,2‐dioleoyl‐3‐phosphatidylethanolamine (DOPE), and 1,2,‐dioleoyl‐3‐phosphatidylserine (DOPS) were added to the system at a ratio of DOPC:DOPE:DOPS = 5:3:2 to mimic a common ER membrane. In addition, 10 1,2‐dioleoyl‐glycerol DAG molecules were randomly distributed in the membrane away from DGAT1. Additionally, we included the acyl‐CoA molecule in the reaction chamber based on the 6VP0 structure (DGAT1^bound^, Figure 2A). For the second system (DGAT1^away^, Figure 2B), we removed the acyl‐CoA molecule in the reaction chamber and placed 10 copies of acyl‐CoA molecules randomly in the cytoplasmic leaflet away from DGAT1. All simulation systems were generated using CHARMM‐GUI *Membrane Builder* [24, 25, 26, 27] and equilibrated by following the *Membrane Builder* standard minimization and equilibration protocols. A total of 150 mM KCl and TIP3P water [28] were added to the bulk region. The temperature was held at 310.15 K by Langevin dynamics, and pressure was maintained at 1 bar under the semi‐isotropic Monte‐Carlo barostat method with a 5 ps^−1^ coupling frequency [29, 30]. All equilibration simulations of 5 μs were performed using OpenMM [31, 32]. Then, the equilibrated systems were converted into Anton2 format to extend the simulation time up to 10 μs [33]. The CHARMM36m force field for protein and lipid was used [34, 35, 36, 37]. For the Anton simulation, NPT ensemble pressure coupling was handled using Martyna–Tobias–Klein barostat with Nose–Hoover temperature coupling to maintain the temperature at 310.5 K [38]. Trajectories are saved based on a 2 fs timestep with 240 ps frame frequency. A 10–12 Å cutoff force‐switching method [39] for the van der Waals interactions was employed, and the particle‐mesh Ewald method [40] was used for long‐range electrostatic interactions. The figures were generated with VMD [41] and PyMOL package.

**TABLE 1:** Simulation systems with acyl‐CoA, DAG, CoA, and TAG.

System	Acyl‐CoA	CoA	DAGs	TAG	Replicas	Time	Phospholipids ratio	
DGAT1^bound^	Bound	X	Unbound	X	1	10 μs	DOPC:DOPE:DOPS	5:3:2
DGAT1^away^	Unbound	X	Unbound	X	1	10 μs		
DGAT1^TAG^	X	Bound	X	Bound	3	5 μs		
DGAT1^TAG‐ABF^	X	Bound	X	Bound	15^a^	100 ns		

^a^ Three different initial conformations were used, with five replica systems for each conformation.

From the DGAT1^bound^ Anton simulation, the last snapshot that contained the close arrangement between DAG's oxygen atoms and acyl‐CoA's sulfur atom was extracted. Through a short minimization using CHARMM [35], the DAG and acyl‐CoA molecules were converted into TAG and CoA molecules. After conversion, a 5 μs equilibration simulation was carried out using OpenMM. As there was no observation of the product egress, three random snapshots from the OpenMM trajectory were chosen, and five replicas of each snapshot were used for adaptive biasing force (ABF) [42] simulations. For the ABF simulation, the CHARMM‐GUI *Enhanced Sampler* [43] module was utilized. A total of 100 ns ABF simulations for 15 replicas were done utilizing NAMD [44] with 0.4 Å bin size and 100 full samples. Two collective variables (CVs) were defined by the distance between the center of mass (COM) of HIS415 in the reaction chamber and either the CoA sulfur atom or a TAG oxygen atom. A value of 20 Å for the lower boundary and lower wall and a value of 90 Å for the upper boundary and upper wall were used with a wall constant of 10 kcal/(mol·Å^2^).

## 3 Results and Discussion

### 3.1 Acyl‐CoA Approaches the Catalytic Reaction Chamber Through TM7–TM8 Opening via Electrostatic Interactions

**FIGURE 3:**
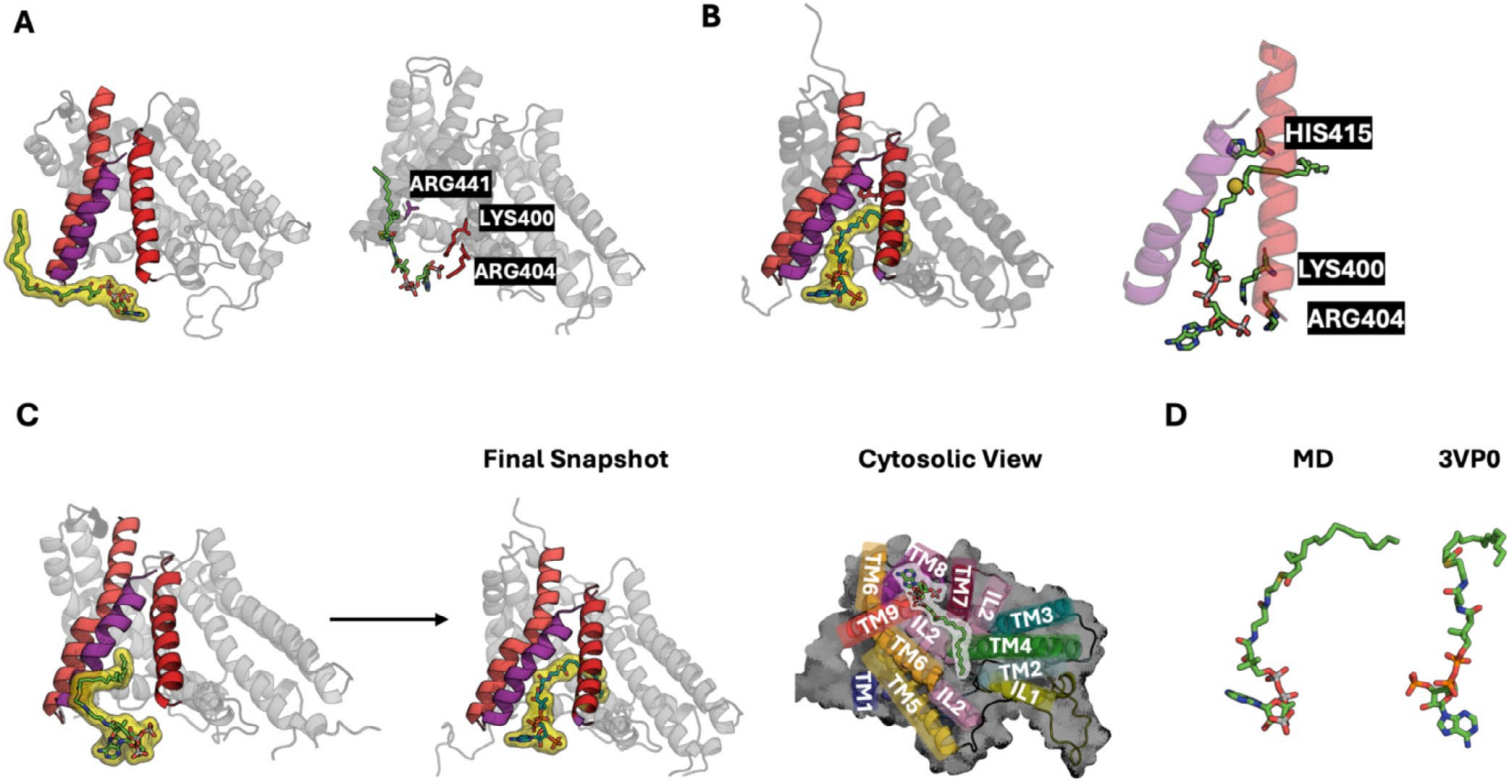
Entry pathway of acyl‐CoA. (A) Initial contact of acyl‐CoA with TM7 (colored in red)‐TM8 (colored in purple) residues (LYS400, ARG404, and ARG441). (B) Interaction between HIS415 with sulfur in acyl‐CoA. (C) Side view of complete entering behavior of acyl‐CoA through the gap between TM7 and TM8. Top view of an entering snapshot from the cytosol side is shown next to the side view. (D) Final conformations of acyl‐CoA from MD simulation and the cryo‐EM structure.

Our initial focus was on investigations of interactions between acyl‐CoA and DGAT1 and how such interactions guide acyl‐CoA's entry toward the reaction chamber. During the 5 μs OpenMM simulation of DGAT1^away^, certain acyl‐CoA molecules ended up at considerable distances from the protein, and others moved closer to the protein complex, either to the vicinity of the reaction chamber or to different parts of the protein. Some acyl‐CoA molecules attempted to enter the catalytic pocket by interacting with TM7 and TM8. In such cases, as acyl‐CoA approached the outer part of the reaction chamber, the acyl‐CoA head group first initiated contact with both TMs (Figure 3A). In particular, the phosphate groups of acyl‐CoA formed strong electrostatic interactions with positively charged residues, such as ARG404 and LYS400 on TM7 and ARG441 on TM8. During the extended Anton simulation, while the acyl‐CoA phosphate groups remained outside interacting with TM7 and TM8, the acyl tail moved in between TM7 and TM8 and then into the reaction chamber, and consequently, its sulfur atom maintained interaction with HIS415 (Figure 3B). Then, the acyl‐CoA head group moved into the reaction chamber through the gap between TM7 and TM8 (Figure 3C). The final snapshot after 10 μs Anton simulation displayed an acyl‐CoA conformation comparable to that of oleoyl‐CoA in the cryo‐EM structure (Figure 3D). It is interesting to note that not a single DAG molecule showed any event to enter the reaction chamber in DGAT1^away^, suggesting the significance of acyl‐tail donor binding before the acceptor substrate (DAG) in DGAT1 in our simulation study.

### 3.2 DAGs Prefer to Enter DGAT1 With Acyl‐CoA Bound in the Reaction Chamber

**FIGURE 4:**
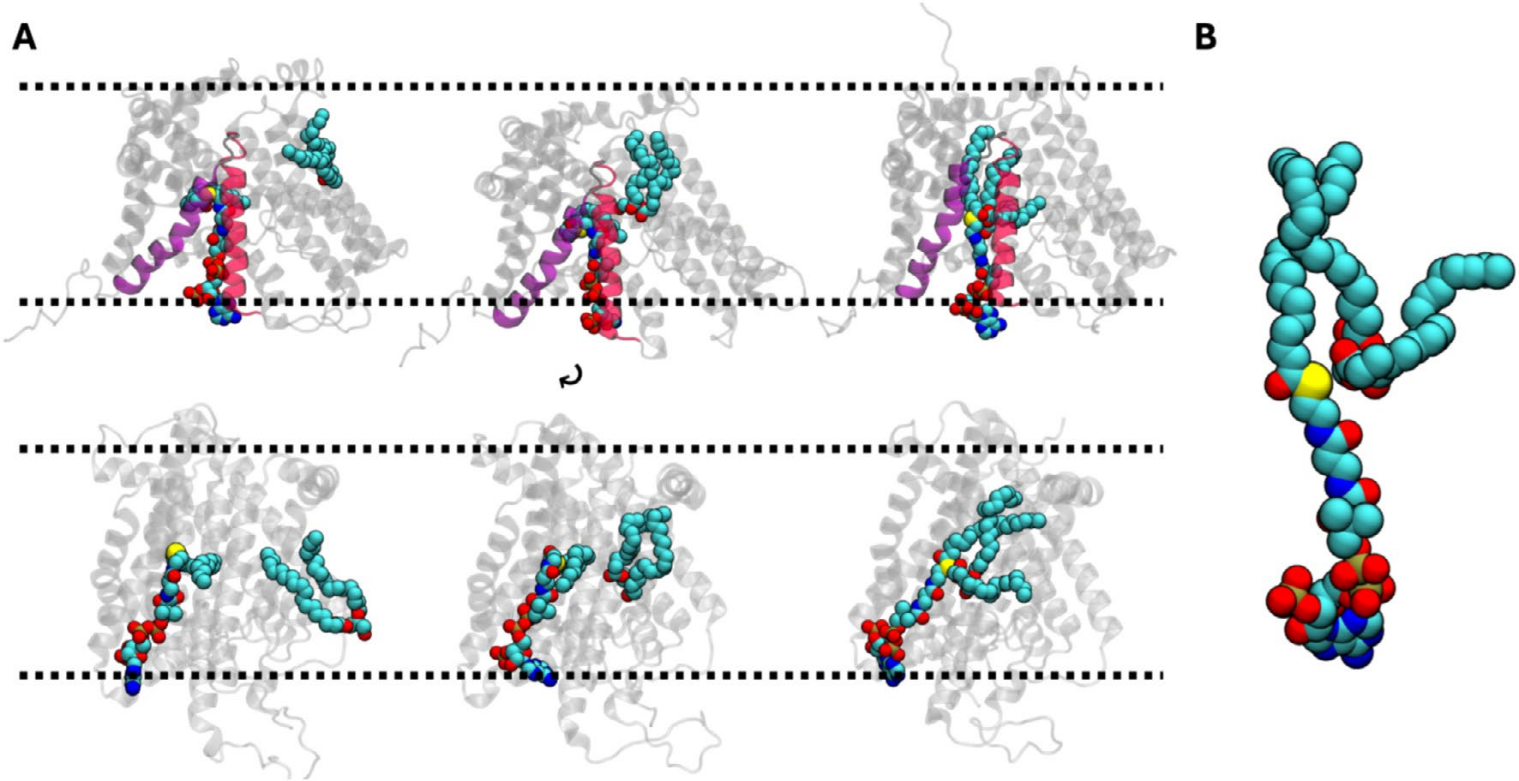
DAG entry pathway. (A) DAG entering snapshots with TM7 (red) and TM8 (purple). The lower panel has a different view for a better representation of the two substrates. The black dotted lines represent the position of the lipid head group in each leaflet. (B) Final conformation of acyl‐CoA and DAG in the reaction chamber.

Next, DAG's entry pathways were explored with the presence of acyl‐CoA in the reaction chamber as in the cryo‐EM structure. During the 5 μs OpenMM simulation of DGAT1^bound^, DAGs moved freely within the membrane (i.e., flip‐flopped). Certain DAG molecules came into closer proximity to the protein, and numerous instances of DAG's attempts to enter the reaction chamber were observed. At 2 μs of the extended Anton simulation, one DAG entered the chamber through a cavity‐like hydrophobic tunnel (we call it a DAG tunnel) made by TM1‐EL1 and TM5 and remained until the end of 10 μs (Figures 4A and S1). The DAG tunnel is positioned on the opposite side of the acyl‐CoA entry route. Starting from the cytosolic side and without a flip‐flop action (Figure 4A), the DAG molecule entered the reaction chamber with an orientation aligning the headgroup oxygen atoms with the acyl‐CoA sulfur atom with the parallel alignment of the tail groups of both substrates (Figure 4B).

### 3.3 Egress Pathways of TAG and CoA Match With the Entry Pathways of DAG and Acyl‐CoA

**FIGURE 5:**
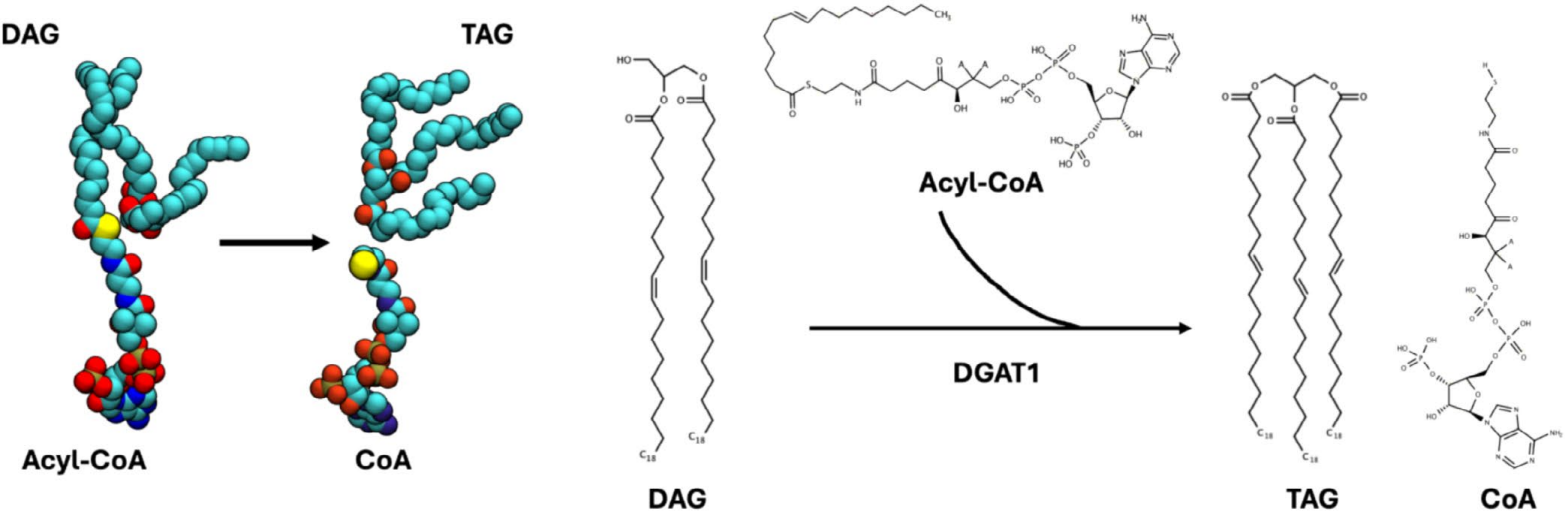
Structure representations of each substrate and product of DGAT1‐mediated catalysis.

In the DGTA1 reaction chamber, one acyl tail is transferred from acyl‐CoA to DAG as part of the protein's acyltransferase activity. After DGAT1 completes its enzymatic activity, the products (TAG and CoA) need to be released from the reaction chamber. To explore their egress pathways, we constructed a simulation system containing CoA and TAG (DGAT1^TAG^ in Table 1) by converting DAG and acyl‐CoA in the reaction chamber to TAG and CoA with a brief minimization as the positions of key atoms (oxygens from DAG and sulfur from acyl‐CoA) were in sufficient proximity (Figure 5).

During the initial 5 μs OpenMM simulation, both TAG and CoA exhibited different conformations inside the catalytic pocket. In most instances, the two products were in close proximity to the interaction between CoA‐sulfur and TAG‐oxygen. In the event of separation, the TAG headgroup shifted away and moved toward the same space in the DAG tunnel, similar to what DAG used in the DGAT1^bound^ simulation. Simultaneously, the CoA rose toward the TM7–TM8 gap through which acyl‐CoA entered the reaction chamber in the DGAT1^away^ simulation. Despite showcasing diverse conformations within the reaction chamber, both products remained inside, making separate attempts to exit from the protein complex. Therefore, to gain more insight into the post‐catalytic egress process, we opted to utilize an ABF method to enhance the sampling by implementing continuous adjustments to free energy along two selected CVs (the distances between the COM of HIS415 in the reaction chamber and either the CoA sulfur atom or a TAG oxygen atom, see also Methods). While ABF is commonly employed for free energy assessments, our focus was on comprehending the substrate's transition within the system. As a result, we chose not to perform free energy calculations in our ABF simulations, since they were not essential to our investigation, which primarily concentrated on analyzing the substrate's behavior and transitions.

Initially, CoA primarily resided near the gap between TM7 and TM8, while the CoA‐headgroup maintained interactions with polar and charged residues on TM7 (THR371, TYR390, LYS400, and ARG404) and TM8 (ALA441, ARG446, and THR371) (Figure S2). Following their exit, CoA moved toward the bulk solvent region rather than the membrane bilayer. Afterward, we observed how TAG exited the reaction chamber using the same tunnel utilized for DAG entry (Figure S1). As TAG left the protein complex, it was released to the bilayer membrane alongside other phospholipids and DAG (Figure S3). During their exit path, the headgroup briefly interacted with GLN292 on TM5, which is closely positioned to TM1, and TYR477 on TM9, located directly behind TM5 (Figure S3). In a different instance, TAG exited by moving directly upward above the membrane bilayer and into the bulk water, rather than integrating into the bilayer with other lipids. This behavior could potentially be an artifact of ABF simulations or a limitation to enhanced sampling methods, which may not always fully capture the true free energy landscape. However, further simulations with no ABF show TAG's integration into the bilayer. To validate the accuracy of our ABF results, additional investigations into the TAG egress pathway are necessary with careful interpretation. Overall, the ABF simulation effectively shows the unique pathways for both products to exit the DGAT1 dimeric complex. Following the catalytic process, an accumulation of TAG within the membrane is known to occur, resulting in the creation of lipid droplets [45, 46].

## 4 Conclusions

Using conventional MD simulation and the ABF enhanced sampling method, we have identified the pathways of two substrates (DAG and acyl‐CoA) and products (TAG and CoA) in DGAT1. Throughout our simulation time, each substrate and product showed a distinctive choice for their entry and egress pathways. Acyl‐CoA accessed the reaction chamber through the TM7–TM8 opening as it approached the protein from the outside of the bilayer membrane on the cytosolic side. Conversely, DAGs attempted to reach the reaction chamber through a different opening located on the opposite side of the protein (TM1‐EL1‐TM5) on the cytosolic side. During the entering process, a single DAG entered the reaction chamber and made contact with bound acyl‐CoA. In the absence of acyl‐CoA, no DAGs managed to enter the protein (during our simulation time), which could indicate the significance of substrate binding order. While the binding of acyl‐CoA did not result in any conformational changes in the protein, it may have an impact on the recruitment of DAGs.

In the previous studies [22, 23], a few key residues were experimentally mutated to test their involvement in DGAT1 enzymatic activities. The point mutation at these residues (THR371, TYR390, LYS400, and ARG404) that are located near the reaction chamber resulted in a moderate reduction of enzymatic activity (30%–70%). Additionally, mutations occurring at residues deeper within the reaction chamber (TRP377, ASN378, HIS382, and SER411) led to a more significant decrease in activity, exceeding 80% reduction. This may indicate that the acyl‐CoA substrate needs to be stabilized during the catalytic process by the residues located within the reaction chamber. Mutations of the residues at the active site, HIS415 and GLU416—both of which are highly conserved in the MBOAT family—resulted in almost complete activity disruptions. Similar results were observed in another study as well. A mutation of the well‐conserved residue, HIS415, to alanine completely abolished enzymatic activity, highlighting its role in the acyl‐transfer process. ASN378, GLN437, and GLN465 mutations to alanine also significantly reduced DGAT1 activity by 50%–75%, demonstrating the functional significance of residues in the reaction chamber. The MET324 mutation also showed a reduced activity, which is known as a residue forming a hydrogen bond with His415 in the absence of the substrate. Furthermore, mutagenesis on VAL381, CYS385, VAL407, and SER411 showed the importance of TM7–TM8 contribution as the DGAT1 activity decreased with the substitution of these residues with larger hydrophobic amino acids.

Clearly, all previous mutation studies were based on the existing structures and mostly focused on the residues around the reaction chamber. We believe that our simulations offer unique opportunities to study the residues that are involved in the substrate entry and product egress pathways. ARG404 and LYS400 on TM7 and ARG441 on TM8 form strong electrostatic interactions with acyl‐CoA and appear to attract it to the TM7–TM8 gap and then to the reaction chamber (Figure 3). These interactions are also involved in the CoA egress (Figure S2). During the DAG entry through the TM1‐EL1‐TM5 tunnel, DAG appears to interact with various residues: TYR111, VAL115, and PRO117 on TM1‐EL1, MET285, ILE296, and TRP340 on TM5, as well as ASN141 on TM2. During its egress pathway, TAG also interacts with similar residues, especially TYR111, LEU114, and VAL115 on TM1‐EL1‐TM2 (Figure S3). It would be interesting to see mutational studies on these residues and their impacts on DGAT1 function.

Since DGAT1's genetic knockout mice studies showed a markedly reduced level of TAG synthesis in various tissues [7, 47, 48], numerous clinical studies were conducted, mostly focusing on pharmacological small molecule inhibitor development [49, 50, 51, 52, 53]. These inhibitors are designed to bind to the active site of DGAT1, thereby blocking its enzymatic activity and reducing TAG synthesis. Despite the progress, the specific molecular processes that lead to the blocking of DGAT1 and TAG production by these substances are not fully understood. In preclinical studies, several DGAT1 inhibitors, such as A922500 [54], PF‐04620110 [55, 56], and T863 [57], have shown potential in reducing TAG levels, indicating their possible use in managing lipid‐related disorders. For example, inhibitor T863 binds to the fatty acid‐CoA binding site of DGAT1, preventing fatty acid entry and reducing TAG production [23]. In a Type 2 diabetic mouse model, Pfizer's PF‐04620110 has been shown to lower blood TAG levels and inflammation by specifically inhibiting DGAT1. However, these inhibitors can have significant side effects. AstraZeneca's AZD7687 [53] caused nausea, vomiting, and diarrhea during clinical trials, while PF‐04620110 led to severe diarrhea and even fatalities in animal testing. Additionally, compounds like Pradigastat [58] and A922500 have been associated with skin issues in mice, including hair loss and sebaceous gland atrophy, suggesting possible long‐term adverse effects. In this context, our study represents a major step forward in this area by presenting a comprehensive visualization of the pathways taken by both substrates and products within the DGAT1 protein complex. This visualization offers important perspectives that may help reveal the details of processes responsible for the observed inhibition, thus opening the door for focused treatment approaches in lipid metabolism disorders. Using extensive MD simulations, we have carefully pinpointed and described distinct entry points and interacting residues for both acyl‐CoA and DAG molecules. This in‐depth exploration of substrate interactions and structural changes not only enhances our comprehension of the intricate architecture of DGAT1, but also provides a valuable understanding of the wider MBOAT protein family. Furthermore, they lay the groundwork for forthcoming advancements that may transform how we investigate and control the functions of MBOAT family members.

## Conflicts of Interest

W.I. is the co‐founder and CEO of MolCube INC. H.L. declares no conflicts of interest.

## Supporting information


**Data S1.** Supporting Information.

## Data Availability

The data that support the findings of this study are available from the corresponding author upon reasonable request.

## References

[1] J. Boyle , “Lehninger Principles of Biochemistry (4th ed.): Nelson, D., and Cox, M,” Biochemistry and Molecular Biology Education 33, no. 1 (2005): 74–75, 10.1002/bmb.2005.494033010419.

[2] A. Cagliari , R. Margis , F. dos Santos Maraschin , A. C. Turchetto‐Zolet , G. Loss , and M. Margis‐Pinheiro , “Biosynthesis of Triacylglycerols (TAGs) in Plants and Algae,” International Journal of Plant Biology 2, no. 1 (2011): e10, 10.4081/pb.2011.e10.

[3] E. P. Kennedy , “Biosynthesis of Complex Lipids,” Federation Proceedings 20 (1961): 934–940. 14455159

[4] S. B. Weiss , E. P. Kennedy , and J. Y. Kiyasu , “The Enzymatic Synthesis of Triglycerides,” Journal of Biological Chemistry 235, no. 1 (1960): 40–44, 10.1016/S0021-9258(18)69581-X. 13843753

[5] S. Cases , S. J. Smith , Y. W. Zheng , et al., “Identification of a Gene Encoding an Acyl CoA:Diacylglycerol Acyltransferase, a Key Enzyme in Triacylglycerol Synthesis,” Proceedings of the National Academy of Sciences of the United States of America 95, no. 22 (1998): 13018–13023, 10.1073/pnas.95.22.13018. 9789033 PMC23692

[6] A. Dahlqvist , U. Ståhl , M. Lenman , et al., “Phospholipid:Diacylglycerol Acyltransferase: An Enzyme That Catalyzes the Acyl‐CoA‐Independent Formation of Triacylglycerol in Yeast and Plants,” Proceedings of the National Academy of Sciences of the United States of America 97, no. 12 (2000): 6487–6492, 10.1073/pnas.120067297. 10829075 PMC18631

[7] S. J. Smith , S. Cases , D. R. Jensen , et al., “Obesity Resistance and Multiple Mechanisms of Triglyceride Synthesis in Mice Lacking Dgat,” Nature Genetics 25, no. 1 (2000): 87–90, 10.1038/75651. 10802663

[8] D. Xu , L. Xie , C. Cheng , F. Xue , and C. Sun , “Triglyceride‐Rich Lipoproteins and Cardiovascular Diseases,” Frontiers in Endocrinology 15 (2024): 1409653, 10.3389/fendo.2024.1409653. 38883601 PMC11176465

[9] J. E. Lambert , M. A. Ramos–Roman , J. D. Browning , and E. J. Parks , “Increased De Novo Lipogenesis Is a Distinct Characteristic of Individuals With Nonalcoholic Fatty Liver Disease,” Gastroenterology 146, no. 3 (2014): 726–735, 10.1053/j.gastro.2013.11.049. 24316260 PMC6276362

[10] N. Chalasani , Z. Younossi , J. E. Lavine , et al., “The Diagnosis and Management of Nonalcoholic Fatty Liver Disease: Practice Guidance From the American Association for the Study of Liver Diseases,” Hepatology 67, no. 1 (2018): 328–357, 10.1002/hep.29367. 28714183

[11] W. P. Esler and K. K. Bence , “Metabolic Targets in Nonalcoholic Fatty Liver Disease,” Cellular and Molecular Gastroenterology and Hepatology 8, no. 2 (2019): 247–267, 10.1016/j.jcmgh.2019.04.007. 31004828 PMC6698700

[12] J. K. Dowman , J. W. Tomlinson , and P. N. Newsome , “Pathogenesis of Non‐Alcoholic Fatty Liver Disease,” QJM 103, no. 2 (2010): 71–83, 10.1093/qjmed/hcp158. 19914930 PMC2810391

[13] C. Chitraju , N. Mejhert , J. T. Haas , et al., “Triglyceride Synthesis by DGAT1 Protects Adipocytes From Lipid‐Induced ER Stress During Lipolysis,” Cell Metabolism 26, no. 2 (2017): 407–418.e3, 10.1016/j.cmet.2017.07.012. 28768178 PMC6195226

[14] B. Deng , W. Kong , X. Shen , et al., “The Role of DGAT1 and DGAT2 in Regulating Tumor Cell Growth and Their Potential Clinical Implications,” Journal of Translational Medicine 22, no. 1 (2024): 290, 10.1186/s12967-024-05084-z. 38500157 PMC10946154

[15] R. Munir , J. Lisec , J. V. Swinnen , and N. Zaidi , “Lipid Metabolism in Cancer Cells Under Metabolic Stress,” British Journal of Cancer 120, no. 12 (2019): 1090–1098, 10.1038/s41416-019-0451-4. 31092908 PMC6738079

[16] C. L. E. Yen , S. J. Stone , S. Koliwad , C. Harris , and R. V. Farese , “Thematic Review Series: Glycerolipids. DGAT Enzymes and Triacylglycerol Biosynthesis,” Journal of Lipid Research 49, no. 11 (2008): 2283–2301, 10.1194/jlr.R800018-JLR200. 18757836 PMC3837458

[17] K. Hofmann , “A Superfamily of Membrane‐Bound O‐Acyltransferases With Implications for Wnt Signaling,” Trends in Biochemical Sciences 25, no. 3 (2000): 111–112, 10.1016/S0968-0004(99)01539-X. 10694878

[18] C. C. Y. Chang , J. Sun , and T. Y. Chang , “Membrane‐Bound O‐Acyltransferases (MBOATs),” Frontiers in Biology 6, no. 3 (2011): 177, 10.1007/s11515-011-1149-z.

[19] J. Yang , M. S. Brown , G. Liang , N. V. Grishin , and J. L. Goldstein , “Identification of the Acyltransferase That Octanoylates Ghrelin, an Appetite‐Stimulating Peptide Hormone,” Cell 132, no. 3 (2008): 387–396, 10.1016/j.cell.2008.01.017. 18267071

[20] P. J. McFie , S. L. Stone , S. L. Banman , and S. J. Stone , “Topological Orientation of Acyl‐CoA:Diacylglycerol Acyltransferase‐1 (DGAT1) and Identification of a Putative Active Site Histidine and the Role of the N Terminus in Dimer/Tetramer Formation,” Journal of Biological Chemistry 285, no. 48 (2010): 37377–37387, 10.1074/jbc.M110.163691. 20876538 PMC2988343

[21] C. E. Coupland , T. B. Ansell , M. S. P. Sansom , and C. Siebold , “Rocking the MBOAT: Structural Insights Into the Membrane Bound O‐Acyltransferase Family,” Current Opinion in Structural Biology 80 (2023): 102589, 10.1016/j.sbi.2023.102589. 37040671

[22] L. Wang , H. Qian , Y. Nian , et al., “Structure and Mechanism of Human Diacylglycerol O‐Acyltransferase 1,” Nature 581, no. 7808 (2020): 329–332, 10.1038/s41586-020-2280-2. 32433610 PMC7255049

[23] X. Sui , K. Wang , N. L. Gluchowski , et al., “Structure and Catalytic Mechanism of a Human Triacylglycerol‐Synthesis Enzyme,” Nature 581, no. 7808 (2020): 323–328, 10.1038/s41586-020-2289-6. 32433611 PMC7398557

[24] E. L. Wu , X. Cheng , S. Jo , et al., “CHARMM‐GUI Membrane Builder Toward Realistic Biological Membrane Simulations,” Journal of Computational Chemistry 35, no. 27 (2014): 1997–2004, 10.1002/jcc.23702. 25130509 PMC4165794

[25] S. Jo , T. Kim , and W. Im , “Automated Builder and Database of Protein/Membrane Complexes for Molecular Dynamics Simulations,” PLoS One 2, no. 9 (2007): e880, 10.1371/journal.pone.0000880. 17849009 PMC1963319

[26] S. Jo , J. B. Lim , J. B. Klauda , and W. Im , “CHARMM‐GUI Membrane Builder for Mixed Bilayers and Its Application to Yeast Membranes,” Biophysical Journal 97, no. 1 (2009): 50–58, 10.1016/j.bpj.2009.04.013. 19580743 PMC2711372

[27] S. Jo , T. Kim , V. G. Iyer , and W. Im , “CHARMM‐GUI: A Web‐Based Graphical User Interface for CHARMM,” Journal of Computational Chemistry 29, no. 11 (2008): 1859–1865, 10.1002/jcc.20945. 18351591

[28] W. L. Jorgensen , J. Chandrasekhar , J. D. Madura , R. W. Impey , and M. L. Klein , “Comparison of Simple Potential Functions for Simulating Liquid Water,” Journal of Chemical Physics 79, no. 2 (1983): 926–935, 10.1063/1.445869.

[29] K. H. Chow and D. M. Ferguson , “Isothermal‐Isobaric Molecular Dynamics Simulations With Monte Carlo Volume Sampling,” Computer Physics Communications 91, no. 1–3 (1995): 283–289, 10.1016/0010-4655(95)00059-O.

[30] J. Åqvist , P. Wennerström , M. Nervall , S. Bjelic , and B. O. Brandsdal , “Molecular Dynamics Simulations of Water and Biomolecules With a Monte Carlo Constant Pressure Algorithm,” Chemical Physics Letters 384, no. 4–6 (2004): 288–294, 10.1016/j.cplett.2003.12.039.

[31] P. Eastman , R. Galvelis , R. P. Peláez , et al., “OpenMM 8: Molecular Dynamics Simulation With Machine Learning Potentials,” Journal of Physical Chemistry B 128, no. 1 (2024): 109–116, 10.1021/acs.jpcb.3c06662. 38154096 PMC10846090

[32] J. Lee , X. Cheng , J. M. Swails , et al., “CHARMM‐GUI Input Generator for NAMD, GROMACS, AMBER, OpenMM, and CHARMM/OpenMM Simulations Using the CHARMM36 Additive Force Field,” Journal of Chemical Theory and Computation 12, no. 1 (2016): 405–413, 10.1021/acs.jctc.5b00935. 26631602 PMC4712441

[33] D. E. Shaw , J. P. Grossman , J. A. Bank , et al., “Anton 2: Raising the Bar for Performance and Programmability in a Special‐Purpose Molecular Dynamics Supercomputer,” in SC14: International Conference for High Performance Computing, Networking, Storage and Analysis (IEEE, 2014), 41–53, 10.1109/SC.2014.9.

[34] B. R. Brooks , C. L. Brooks , A. D. Mackerell , et al., “CHARMM: The Biomolecular Simulation Program,” Journal of Computational Chemistry 30, no. 10 (2009): 1545–1614, 10.1002/jcc.21287. 19444816 PMC2810661

[35] B. R. Brooks , R. E. Bruccoleri , B. D. Olafson , D. J. States , S. Swaminathan , and M. Karplus , “CHARMM: A Program for Macromolecular Energy, Minimization, and Dynamics Calculations,” Journal of Computational Chemistry 4, no. 2 (1983): 187–217, 10.1002/jcc.540040211.

[36] J. B. Klauda , R. M. Venable , J. A. Freites , et al., “Update of the CHARMM All‐Atom Additive Force Field for Lipids: Validation on Six Lipid Types,” Journal of Physical Chemistry B 114, no. 23 (2010): 7830–7843, 10.1021/jp101759q. 20496934 PMC2922408

[37] J. Huang , S. Rauscher , G. Nawrocki , et al., “CHARMM36m: An Improved Force Field for Folded and Intrinsically Disordered Proteins,” Nature Methods 14, no. 1 (2016): 71–73, 10.1038/nmeth.4067. 27819658 PMC5199616

[38] R. A. Lippert , C. Predescu , D. J. Ierardi , et al., “Accurate and Efficient Integration for Molecular Dynamics Simulations at Constant Temperature and Pressure,” Journal of Chemical Physics 139, no. 16 (2013): 164106, 10.1063/1.4825247. 24182003

[39] P. J. Steinbach and B. R. Brooks , “New Spherical‐Cutoff Methods for Long‐Range Forces in Macromolecular Simulation,” Journal of Computational Chemistry 15, no. 7 (1994): 667–683, 10.1002/jcc.540150702.

[40] U. Essmann , L. Perera , M. L. Berkowitz , T. Darden , H. Lee , and L. G. Pedersen , “A Smooth Particle Mesh Ewald Method,” Journal of Chemical Physics 103, no. 19 (1995): 8577–8593, 10.1063/1.470117.

[41] W. Humphrey , A. Dalke , and K. Schulten , “VMD: Visual Molecular Dynamics,” Journal of Molecular Graphics 14, no. 1 (1996): 33–38, 10.1016/0263-7855(96)00018-5. 8744570

[42] E. Darve , D. Rodríguez‐Gómez , and A. Pohorille , “Adaptive Biasing Force Method for Scalar and Vector Free Energy Calculations,” Journal of Chemical Physics 128, no. 14 (2008): 144120, 10.1063/1.2829861. 18412436

[43] D. Suh , S. Feng , H. Lee , et al., “CHARMM‐GUI Enhanced Sampler for Various Collective Variables and Enhanced Sampling Methods,” Protein Science 31, no. 11 (2022): e4446, 10.1002/pro.4446. 36124940 PMC9601830

[44] J. C. Phillips , D. J. Hardy , J. D. C. Maia , et al., “Scalable Molecular Dynamics on CPU and GPU Architectures With NAMD,” Journal of Chemical Physics 153, no. 4 (2020): 044130, 10.1063/5.0014475. 32752662 PMC7395834

[45] R. W. Klemm and P. Carvalho , “Lipid Droplets Big and Small: Basic Mechanisms That Make Them All,” Annual Review of Cell and Developmental Biology 40, no. 1 (2024): 143–168, 10.1146/annurev-cellbio-012624-031419. 39356808

[46] A. R. Thiam and E. Ikonen , “Lipid Droplet Nucleation,” Trends in Cell Biology 31, no. 2 (2021): 108–118, 10.1016/j.tcb.2020.11.006. 33293168

[47] C. Chitraju , A. W. Fischer , Y. A. Ambaw , et al., “Mice Lacking Triglyceride Synthesis Enzymes in Adipose Tissue Are Resistant to Diet‐Induced Obesity,” eLife 12 (2023): RP88049, 10.7554/eLife.88049. 37782317 PMC10545428

[48] H. C. Chen , S. J. Smith , Z. Ladha , et al., “Increased Insulin and Leptin Sensitivity in Mice Lacking Acyl CoA:Diacylglycerol Acyltransferase 1,” Journal of Clinical Investigation 109, no. 8 (2002): 1049–1055, 10.1172/JCI14672. 11956242 PMC150948

[49] H. Denison , C. Nilsson , M. Kujacic , et al., “Proof of Mechanism for the DGAT1 Inhibitor AZD7687: Results From a First‐Time‐in‐Human Single‐Dose Study,” Diabetes, Obesity and Metabolism 15, no. 2 (2013): 136–143, 10.1111/dom.12002. 22950654

[50] M. Okour , A. Gress , X. Zhu , D. Rieman , J. D. Lickliter , and R. A. Brigandi , “First‐in‐Human Pharmacokinetics and Safety Study of GSK3008356, a Selective DGAT1 Inhibitor, in Healthy Volunteers,” Clinical Pharmacology in Drug Development 8, no. 8 (2019): 1088–1099, 10.1002/cpdd.691. 30950565

[51] B. S. Maciejewski , J. L. LaPerle , D. Chen , et al., “Pharmacological Inhibition to Examine the Role of DGAT1 in Dietary Lipid Absorption in Rodents and Humans,” American Journal of Physiology. Gastrointestinal and Liver Physiology 304, no. 11 (2013): G958–G969, 10.1152/ajpgi.00384.2012. 23558010

[52] C. D. Meyers , A. Amer , T. Majumdar , and J. Chen , “Pharmacokinetics, Pharmacodynamics, Safety, and Tolerability of Pradigastat, a Novel Diacylglycerol Acyltransferase 1 Inhibitor in Overweight or Obese, but Otherwise Healthy Human Subjects,” Journal of Clinical Pharmacology 55, no. 9 (2015): 1031–1041, 10.1002/jcph.509. 25854859

[53] H. Denison , C. Nilsson , L. Löfgren , et al., “Diacylglycerol Acyltransferase 1 Inhibition With AZD7687 Alters Lipid Handling and Hormone Secretion in the Gut With Intolerable Side Effects: A Randomized Clinical Trial,” Diabetes, Obesity and Metabolism 16, no. 4 (2014): 334–343, 10.1111/dom.12221. 24118885

[54] A. J. King , J. A. Segreti , K. J. Larson , et al., “In Vivo Efficacy of Acyl CoA: Diacylglycerol Acyltransferase (DGAT) 1 Inhibition in Rodent Models of Postprandial Hyperlipidemia,” European Journal of Pharmacology 637, no. 1–3 (2010): 155–161, 10.1016/j.ejphar.2010.03.056. 20385122

[55] R. L. Dow , J. C. Li , M. P. Pence , et al., “Discovery of PF‐04620110, a Potent, Selective, and Orally Bioavailable Inhibitor of DGAT‐1,” ACS Medicinal Chemistry Letters 2, no. 5 (2011): 407–412, 10.1021/ml200051p. 24900321 PMC4018057

[56] R. L. Dow , M. Andrews , G. E. Aspnes , et al., “Design and Synthesis of Potent, Orally‐Active DGAT‐1 Inhibitors Containing a Dioxino[2,3‐d]Pyrimidine Core,” Bioorganic & Medicinal Chemistry Letters 21, no. 20 (2011): 6122–6125, 10.1016/j.bmcl.2011.08.028. 21908190

[57] J. Cao , Y. Zhou , H. Peng , et al., “Targeting Acyl‐CoA:Diacylglycerol Acyltransferase 1 (DGAT1) With Small Molecule Inhibitors for the Treatment of Metabolic Diseases,” Journal of Biological Chemistry 286, no. 48 (2011): 41838–41851, 10.1074/jbc.M111.245456. 21990351 PMC3308890

[58] D. Bauer , R. L. Soon , K. Kulmatycki , et al., “The DGAT1 Inhibitor Pradigastat Does Not Induce Photosensitivity in Healthy Human Subjects: A Randomized Controlled Trial Using Three Defined Sunlight Exposure Conditions,” Photochemical & Photobiological Sciences 15, no. 9 (2016): 1155–1162, 10.1039/c6pp00042h. 27471837

