# Supplementary material for: Substrates (Acyl‐CoA and Diacylglycerol) Entry and Products (CoA and Triacylglycerol) Egress Pathways in DGAT1
Source: J Comput Chem. 2025 Apr 19;46(11):e70108. doi: 10.1002/jcc.70108 (PMC12008735; doi:10.1002/jcc.70108)
Supplement: Supplementary file 1 — Data S1. Supporting Information. [file JCC-46-0-s001.pdf]

Supporting Information

Substrates (Acyl-CoA and diacylglycerol) Entry and Products (CoA and triacylglycerol) Egress Pathways in DGAT1

*Hwayoung Lee and Wonpil Im**

Department of Biological Sciences, Lehigh University, Bethlehem, PA 18015, USA

*Corresponding author: wonpil@lehigh.edu

KEYWORDS: DGAT1, Diacylglycerol, Acyl-CoA, Diacylglycerol O-acyltransferase 1, MBOAT, Triacylglycerol

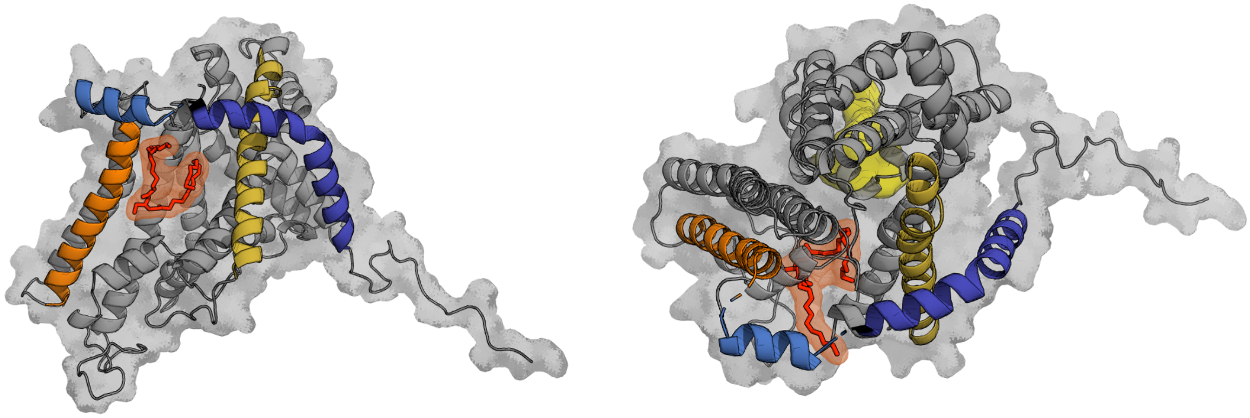

Figure S1. DAG preferred entrance in DGAT1. (Left) DAG approaches through the lateral gap between TM1 (colored in dark blue)-EL1 (colored in light blue)-TM2 (colored in orange)-TM5 (colored in ivory) when acyl-CoA is bound to the reaction chamber. (Right) DAG's approach is always observed through this gap that is located at the opposite side of the acyl-CoA's binding site (depicted in yellow surface representation) near the reaction chamber.

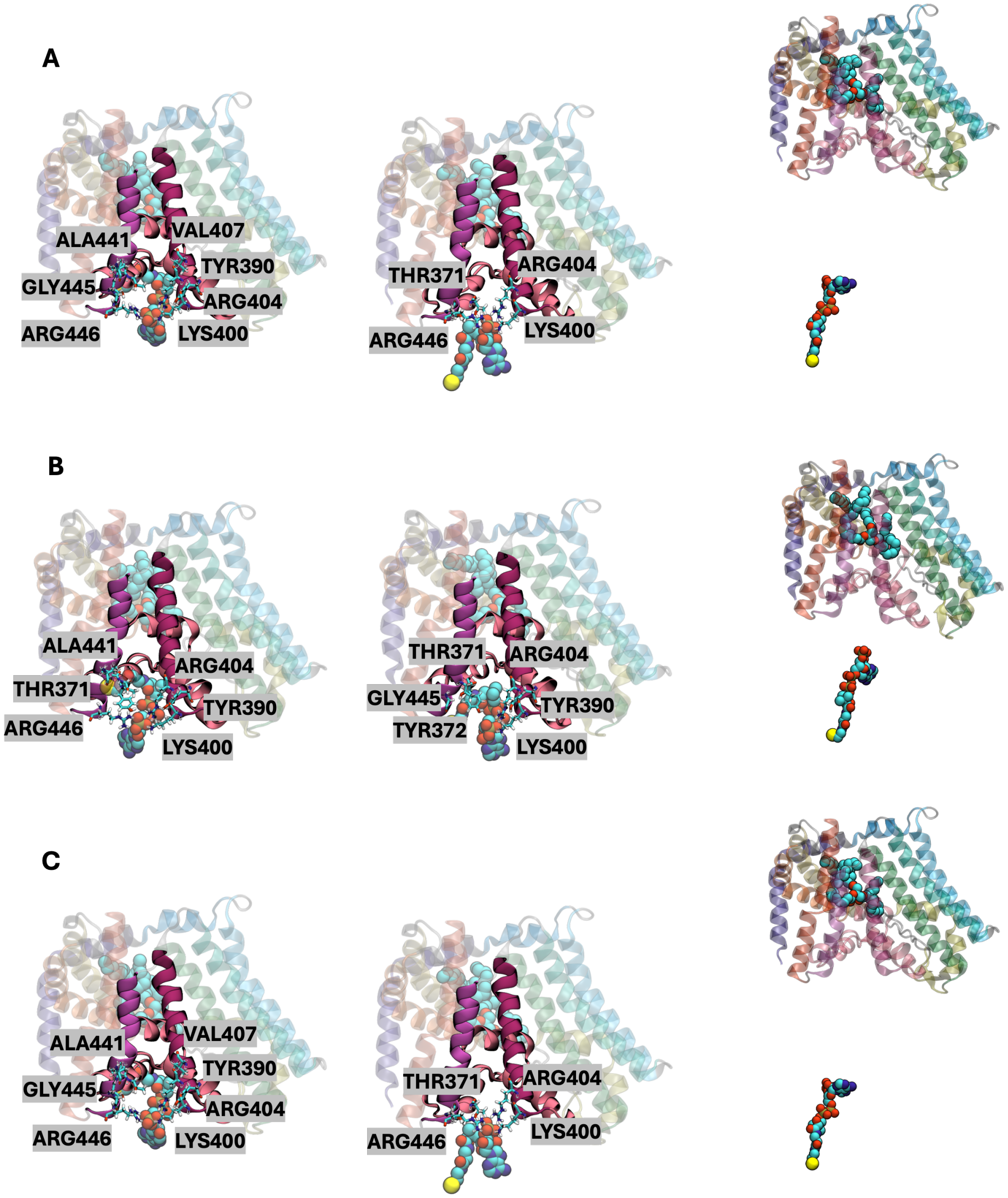

Figure S2. Egress pathways of CoA. (A-C) Contacting residues are marked with licorice representation with residue names. Each figure represents different replica systems, starting with initial conformation (left) to the exiting point (right).

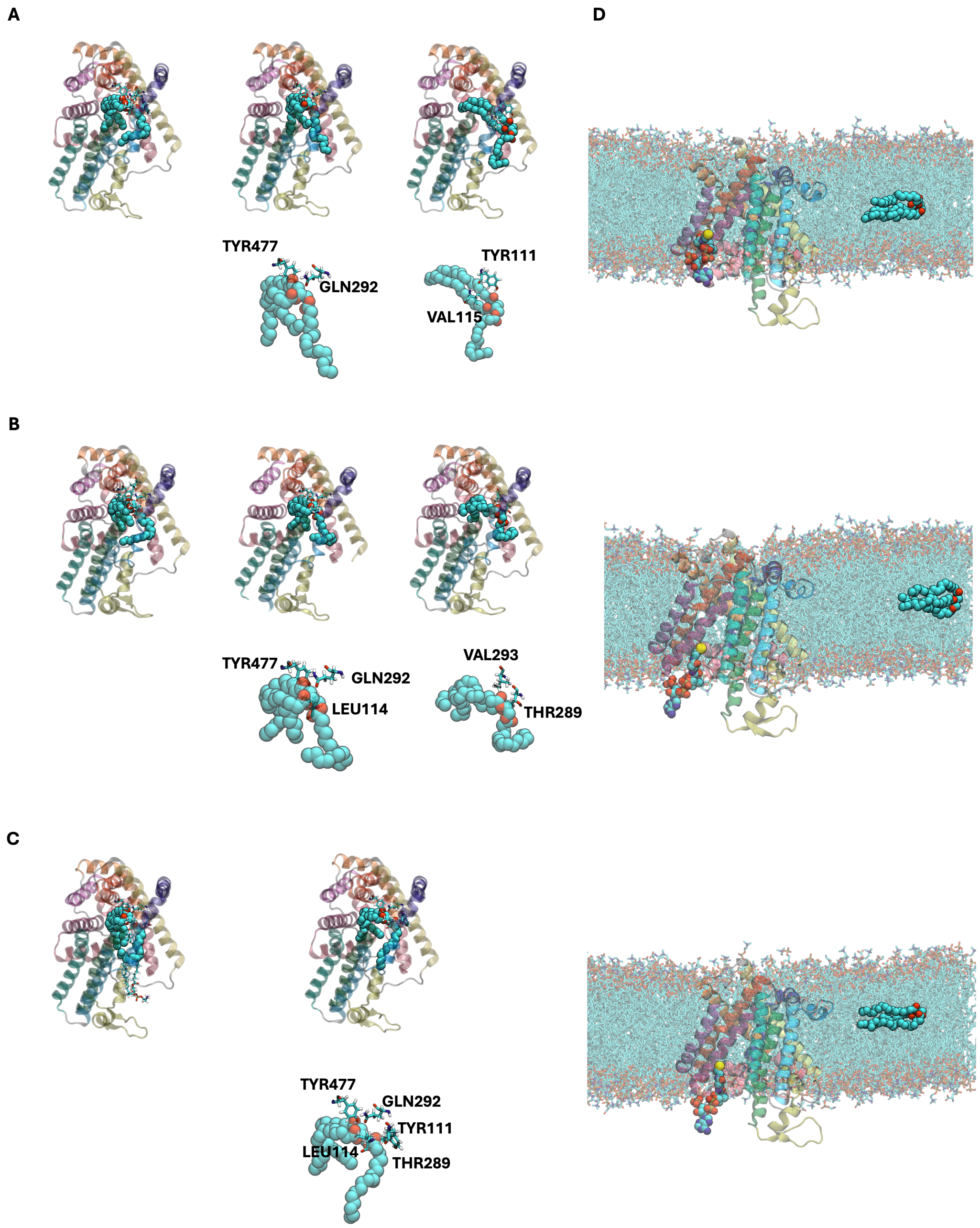

Figure S3. Egress pathways of TAG. (A-C) Contacting residues are marked with licorice representation with residue names. Each figure represents different replica systems, starting with initial conformation (left) to the point of exit (right). (D) After TAG exited, it joined the bilayer membrane and stayed in the bilayer center.
